# Paramagnetic Beads and Magnetically Mediated Strain Enhance Cardiomyogenesis in Mouse Embryoid Bodies

**DOI:** 10.1371/journal.pone.0113982

**Published:** 2014-12-12

**Authors:** Laura R. Geuss, Douglas C. Wu, Divya Ramamoorthy, Corinne D. Alford, Laura J. Suggs

**Affiliations:** 1 The University of Texas at Austin, Institute of Cell and Molecular Biology, Austin, Texas, United States of America; 2 The University of Texas at Austin, Department of Biomedical Engineering, Austin, Texas, United States of America; University of California, San Diego, United States of America

## Abstract

Mechanical forces play an important role in proper embryologic development, and similarly such forces can directly impact pluripotency and differentiation of mouse embryonic stem cells (mESC) *in vitro*. In addition, manipulation of the embryoid body (EB) microenvironment, such as by incorporation of microspheres or microparticles, can similarly influence fate determination. In this study, we developed a mechanical stimulation regimen using permanent neodymium magnets to magnetically attract cells within an EB. Arginine-Glycine-Aspartic Acid (RGD)-conjugated paramagnetic beads were incorporated into the interior of the EBs during aggregation, allowing us to exert force on individual cells using short-term magnetization. EBs were stimulated for one hour at different magnetic field strengths, subsequently exerting a range of force intensity on the cells at different stages of early EB development. Our results demonstrated that following exposure to a 0.2 Tesla magnetic field, ESCs respond to magnetically mediated strain by activating Protein Kinase A (PKA) and increasing phosphorylated extracellular signal-regulated kinase 1/2 (pERK1/2) expression. The timing of stimulation can also be tailored to guide ESC differentiation: the combination of bone morphogenetic protein 4 (BMP4) supplementation with one hour of magnetic attraction on Day 3 enhances cardiomyogenesis by increasing contractile activity and the percentage of sarcomeric α-actin-expressing cells compared to control samples with BMP4 alone. Interestingly, we also observed that the beads alone had some impact on differentiation by increasingly slightly, albeit not significantly, the percentage of cardiomyocytes. Together these results suggest that magnetically mediated strain can be used to enhance the percentage of mouse ESC-derived cardiomyocytes over current differentiation protocols.

## Introduction

Myocardial Infarction (MI) is one of the most prevalent diseases in America, accounting for approximately 50% (7.6 million) of the 15.4 million Americans suffering from coronary heart disease [1]. As an alternative to invasive surgical treatment options, cell therapy holds promise in promoting recovery following heart failure. For this strategy to be effective in a clinical setting, an adequate cardiomyocyte cell source must be identified. Differentiation of cardiomyocytes from progenitor cells such as pluripotent stem cells (PSCs) has the most potential to derive a large enough population to be a clinically relevant source. The most effective differentiation regimens include the addition of bone morphogenetic protein 4 (BMP4) to mouse embryonic stem cells (mESCs), as well as the supplementation of basic Fibroblast Growth Factor (bFGF), Activin, and Wnt to human embryonic stem cell (hESC) cultures [2]. While the highest purity of the differentiated cardiomyocyte population is generated from ESC monolayer culture (up to 90–95% cardiomyocytes from hESCs) [3], establishing a protocol to efficiently differentiate ESCs within an embryoid body (EB) could potentially increase the final cell yield, which is a bottleneck for the clinical use of ESC-derived cardiomyocytes. To date, the highest cardiomyocyte yield from murine EB culture is approximately 20% [4].

Mechanical stimulation has been well demonstrated to direct differentiation of many progenitor cell types, including ESCs. A variety of stimulation regimens have been used to differentiate PSCs into cardiomyocytes, the most common being fluid shear and cyclic strain [5]. In general, the onset and duration of stimulation can have the most profound effects on cardiomyogenesis. Short durations of cyclic strain in Day 4–6 mouse EBs increase the expression of Connexin 43 and mesodermal marker NKX2.5 [6]–[8], while exposure to strain at later time points (after Day 9) can inhibit mESC differentiation [9]. In hESC monolayers, earlier exposure (Day 1 through 4) has been demonstrated to increase α-actinin expression and alignment [10]. In Day 1 mouse ESC monolayers, as little as 30 minutes exposure to 10 dyn/cm^2^ shear stress increases cardiovascular marker expression compared to controls [11]. Similar magnitudes have also been effective on Day 3 EBs attached to tissue culture plastic [12].

Magnetic Twisting Cytometry (MTC) is another stimulation technique that has been widely used to study mechanotransduction in cells [13]–[15]. This technique employs Arginine-Glycine-Aspartic Acid (RGD)-coated paramagnetic beads, which when attached to the cell surface via integrins induce strain on the cell during magnetization. MTC has recently been used to demonstrate that integrin-mediated forces can decrease pluripotency in mESCs [16], [17]. In these experiments, single mESCs were plated and attached to RGD-Beads. The cells were exposed to an oscillatory stress (17.5 Pa and 0.3 Hz) for one hour, and changes in pluripotency marker expression were examined over time. The authors discovered that only an hour of stimulation was required to significantly decrease OCT3/4 24 hours after exposure. These results demonstrated that pluripotency in ESCs can be linked to integrin-RGD interactions and manipulation; however, the effects on terminal differentiation are still not well understood.

A challenge with mechanical stimulation of EBs is obtaining homogenous exposure of all cells within the EB to a similar magnitude of stress. In addition, few studies have compared the combinatorial effect of biochemical supplementation and mechanical stimulation. In this study, we hypothesized that magnetization of RGD-immobilized beads within an EB can activate stress-dependent signaling pathways in ESCs, and subsequently increase the population of cardiomyocytes. We have adapted the MTC-stimulation regimen by loading mouse EBs with RGD-beads and have examined the effect of magnetization at the earliest stages of EB development. Here, we report that magnetic strain applied through paramagnetic beads has the potential to direct lineage commitment in EB, and that the terminal phenotype is influenced by the onset of magnetic stimulation.

## Materials/Methods

### Embryonic Stem Cell Culture

R1 mouse ESCs (A. Nagy, Toronto, Canada) were expanded feeder-free on 0.1% gelatin-coated cell culture flasks (Stem Cell Technologies, Vancouver, Canada) in Growth Medium containing Knockout Dulbecco's Modified Eagle's Medium (Invitrogen, Grand Island, NY) supplemented with 15% ES-Cult Fetal Bovine Serum (Stem Cell Technologies), 1% Glutamax (Invitrogen), 1% Non-Essential Amino Acids (Invitrogen), 1% Nucleosides (Millipore, Billerica, MA), 1% Penicillin/Streptomycin (Invitrogen), 0.1 mM β-Mercaptoethanol (Invitrogen) and 10^3^ U/ml Leukemia Inhibitory Factor (LIF; Millipore). Medium was changed in full each day, and cells were passaged every 2 days before reaching approximately 70% confluence.

### Bead Preparation

In these experiments, 1 µm BcMag tosyl-activated paramagnetic beads (Bioclone, San Diego, CA) were coated with Peptite 2000 (Advanced Biomatrix, San Diego, CA) using previously established methods [13], [15], [18], [19]. Briefly, 50 µg Peptite 2000 was bound to 1 mg of paramagnetic beads for 72 hours at 4°C in 0.1 M Carbonate Buffer, pH 9 as suggested by the manufacturer. Acetylated-Low Density Lipoprotein (AcLDL, Biomedical Technologies, Stoughton, MA), which does not bind integrins or support stress transfer across the cell membrane, [18] was used as a negative control and immobilized to the beads at the same concentration (50 µg protein/mg beads). The protein-bound beads were rinsed three times in PBS containing calcium and magnesium with a Dynal magnet (Invitrogen) and blocked for 1 hour in PBS containing 5% Bovine Serum Albumin (Sigma-Aldrich, St. Louis, MO). Following an additional three rinses in PBS, the beads were resuspended in medium and added to the Aggrewell 400 plates at 1 mg beads per well.

### Embryoid Body Formation and Bead Incorporation

ESCs were enzymatically released from the gelatin-coated plates with trypsin/EDTA (ATCC, Manassas, VA) and resuspended in Differentiation Medium (Growth Medium without LIF). Approximately 1.2 million cells were added to each well of a 24-well Aggrewell 400 plate (Stem Cell Technologies) as directed by the manufacturer. The final EB sizes were approximately 1,000 cells/EB. Once the ESCs were allowed to settle for 30 minutes at 37°C/5%CO_2_, the beads were distributed to the appropriate wells and collected into the bottom of the Aggrewells using magnets, ensuring that beads were homogenously distributed among the ESCs as they aggregated. The EBs were kept in the Aggrewell 400 plates for 3 days, and medium was partially changed daily. Day 3 EBs were transferred to suspension culture in ultra-low attachment plates and medium was changed in full every 2 days. In the BMP4-treated group, 10 ng/ml BMP4 was added from Days 1 through 7 as described previously [20]. On Day 7, the EBs were allowed to attach to gelatin-coated plates and maintained for up to 18 days at 37°C/5%CO_2_.

### EB viability

EB viability was evaluated on Day 2 and Day 7 with a live/dead viability kit (Life Technologies) as described previously with a few modifications [21]. At each timepoint, the EBs were collected and rinsed in PBS containing calcium and magnesium and incubated in 2 µM calcein-AM and 4 µM ethidium homodimer diluted in PBS for 30 minutes at 37°C on an end-to-end shaker. The EBs were imaged using a Zeiss scanning confocal microscope. Live and dead cells were labeled with calcein-AM (green) and ethidium homodimer (red), respectively.

### β1 integrin inhibition

For β1 integrin blocking experiments, ESCs were trypsinized and incubated in 500 µg/ml Gly-Arg-Gly-Asp-Ser (GRGDS; Anaspec Inc, Fremont, CA) in serum-free growth medium containing 10^3^ U/ml LIF to prevent aggregation. The ESCs were incubated in ultra-low attachment plates at 37°C/5%CO_2_ for 4 hours with gentle shaking. Immediately following incubation, the ESCs were aggregated using Aggrewell 400 plates as described previously.

### Spontaneous contractile activity

The presence of contractile areas in EBs was assessed as described previously [20], [22]. After 7 days of suspension culture, EBs were transferred to a 0.1% gelatin-coated 48-well plate using a 1 ml wide bore tip. Approximately 1–2 EBs were added to each well for a final count of 10–15 EBs per group. The EBs were monitored for 10 days after plating, with 70% medium changes every 2 days. At each timepoint, the percentage of EBs with contractile areas was calculated relative to the total number of EBs per group.

### Flow Cytometry

EBs were collected on Day 18 to measure sarcomeric α-actin as described previously with a few modifications [22]. Following dissociation in Accumax (Stem Cell Technologies), the ESCs were incubated in anti-mouse sarcomeric α-actin (5c5, Sigma-Aldrich; 0.2 µl/100 µl reaction) for 45 minutes at room temperature in the dark with rotation. Mouse IgM isotype controls (Stem Cell Biotechnology) were included. Following another three rinses, the cells were incubated with goat anti-mouse IgM FITC (Stem Cell Biotechnology) for 30 minutes in the dark. The ESCs were resuspended in 500 µl cold PBS at a final concentration of 1×10^6^ cells/ml. The number of sarcomeric α-actin positive cells was analyzed using an Accuri C6 Flow Cytometer (BD Biosciences) to capture a minimum of 10,000 events. Positive gates were set at 1% of the isotype control population. Analysis was performed using FlowJo software (Tree Star Inc, Ashland, OR).

### SDS-Page and Western Blotting

Immediately following magnetization, EBs were collected from Aggrewell 400 plates, rinsed in PBS, and disrupted in cold RIPA buffer (Santa Cruz Biotechnology, Santa Cruz, CA). The suspension was centrifuged for 10 minutes at 10,000xg at 4°C. The supernatant was transferred to a 10 kDa MWCO filter unit (Millipore, Billerica, MA) and centrifuged at 10,000xg for 4 minutes at room temperature to concentrate the protein fraction. The protein was then inverted into a new tube and collected at 1,000xg for 2 minutes at room temperature. Total protein was analyzed by BCA assay (Thermo Scientific), and 10 µg protein was loaded into each lane of a 10% Mini-Protean TGX precast gel (Bio-rad, Hercules, CA). Following western transfer, the PVDF membranes were blocked for 1 hour in 5% Milk in TBST (Tris Buffered Saline with 0.05% Tween-20) and incubated overnight at 4°C in rabbit anti-cAMP protein kinase catalytic subunit antibody (Abcam, 1∶3000), rabbit anti-pERK1/2 antibody (Cell Signaling Technology, 1∶400) or rabbit anti-ERK1/2 (Cell Signaling Technology, 1∶1000) diluted in blocking buffer. After rinsing, the blots were incubated for 1 hour at room temperature in goat-anti rabbit HRP antibody (Santa Cruz Biotechnology, 1∶1000). The signal was developed using SuperSignal West Pico Substrate (Thermo Scientific) as directed by the manufacturer, and images were obtained with a FluorChem HD2 digital imager (Protein Simple, Santa Clara, CA). Band intensity was calculated and analyzed using AlphaView software (Protein Simple). The data represent the results of protein isolated from at least three independent experiments.

### Histology and Immunostaining

EBs were fixed with 4% buffered formalin, rinsed in PBS, and resuspended in OCT solution (Thermo Scientific) for cryosectioning. Frozen sections (10 µm) were rehydrated for 10 minutes in PBS. Prussian Blue staining was performed to locate the beads within the EBs. The sections were incubated in a 1∶1 solution of 20% aqueous hydrochloric acid: 10% aqueous potassium ferrocyanide for 20 minutes at room temperature. Following three rinses in distilled water, the slides were counterstained in Nuclear Fast Red (Sigma-Aldrich) for 5 minutes at room temperature. The slides were rinsed twice in distilled water, cleared and mounted with Cytoseal (Thermo Scientific).

For immunostaining, epitope retrieval was performed using 0.25% trypsin solution (Thermo Scientific) for 20 minutes at 37°C in a humidified chamber. To detect intracellular protein, samples were permeabilized in 0.5% Triton-X in PBS for 10 minutes at room temperature. After rinsing, sections were blocked for 1 hour at room temperature in 10% normal goat serum (NGS) or normal donkey serum (NDS). The samples were incubated overnight at 4°C in rat anti-integrin β1 (Abcam, Cambridge, MA, 1∶100 in 1% NDS), mouse anti-sarcomeric α-actin (5c5, Sigma Aldrich, 1∶500 in 1% NGS) or rabbit anti-connexin 43 (Cx43, Cell Signaling Technologies, 1∶250 in 1% NGS). The sections were rinsed in TBST and incubated in goat anti-mouse IgM FITC (Abcam, 1∶100 in 1% NGS), goat anti-rabbit Alexa Fluor 488 (Invitrogen, 1∶500 in 1% NGS) or donkey anti-rat Alexa Fluor 594 (Invitrogen, 1∶500 in 1% NDS) for 1 hour at room temperature. The samples were counterstained with 5 µg/ml 4',6-diamidino-2-phenylindole (DAPI, Life Technologies) for 15 minutes and mounted with Prolong Gold antifade reagent (Life Technologies). Beta 1 integrin images were taken using a Leica DMI2000B microscope with a Leica DFC290 camera. Cx43 and sarcomeric α-actin images were taken using a Zeiss scanning confocal microscope.

### Statistics

Statistical analysis was performed using StatPlus LE software (AnalystSoft). All groups analyzed represent three independent experiments. Data from each of the groups was compared using one-way ANOVA coupled with Tukey's Post Hoc test. Values are reported as mean ± standard deviation unless indicated otherwise. *p*-values less than 0.05 were considered statistically significant.

## Results

### Paramagnetic beads do not affect EB morphology

**Figure 1:**
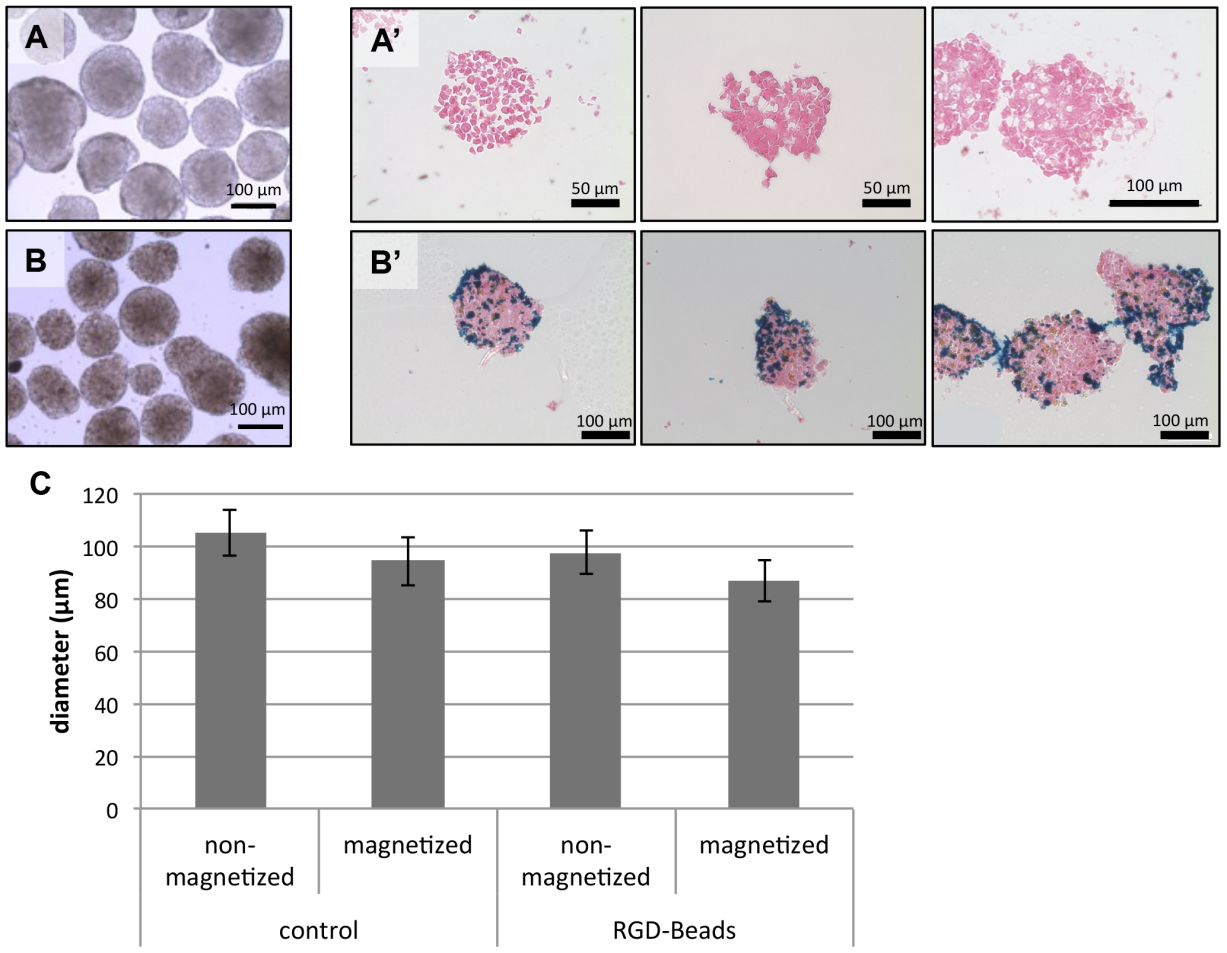
Gross morphology of EBs with immobilized beads. Bead incorporation was confirmed by light micrographs (A–B) and Prussian Blue staining (A′–B′). (C) Size comparison of EBs with or without magnetization.

To prepare the EBs, we used Aggrewell 400 plates to aggregate the ESCs and also facilitate bead incorporation within the aggregate. By FACS analysis, we calculated that the approximate number of beads loaded into each EB was 500 beads/EB (data not shown). We confirmed the bead incorporation procedure by light microscopy and Prussian Blue staining on sectioned EBs, in which the latter stains ferric ions a blue color. In comparison to the control EBs with no beads (Fig. 1 A, A′), the EBs with the magnetic beads were darker in light micrographs (Fig. 1B), and the beads appeared distributed throughout the entire EB (Fig. 1B′). To examine the effects of beads or magnetization on EB gross morphology, we quantified EB size 24 hours after magnetic attraction. No significant differences were observed regardless of bead incorporation or magnetization (Fig. 1C).

### Magnetic field strength proportionally increases force on cells by RGD-Beads

**Figure 2:**
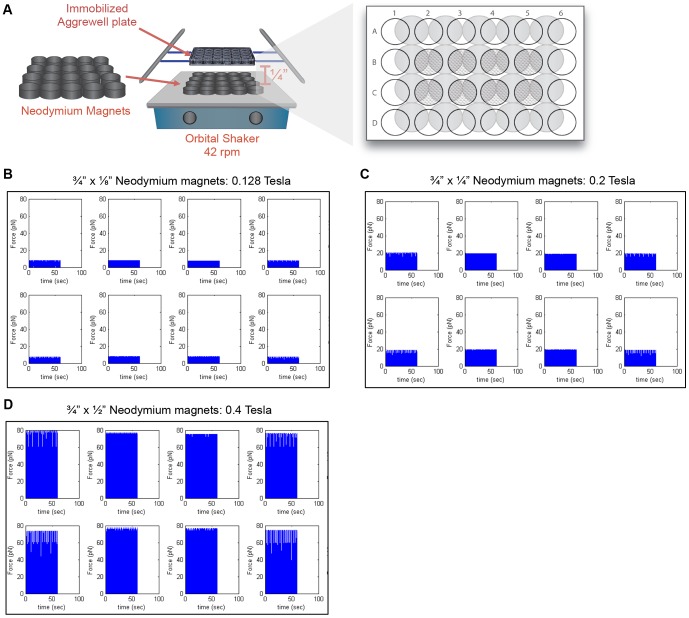
Magnetic attraction apparatus setup. (A) An array of N42 permanent neodymium magnets with different thicknesses were arranged on an orbital shaker. The force applied to the cells was modeled using a customized Matlab program over the course of 1 min (42 cycles) at 0.128 Tesla (B), 0.2 Tesla (C), and 0.4 Tesla (D).

In this study, we used RGD-immobilized paramagnetic beads to exert mechanical stimuli to cells within the interior of EBs. As mentioned previously, Uda *et al* (2011) demonstrated that one hour of MTC using RGD-beads decreased pluripotency markers in mESCs [16]. In this study, we investigated how magnetization at different field strengths can affect mechanotransduction in murine EBs. We had previously observed that cycling neodymium magnets underneath EBs resulted in a continuous attraction of the EBs to the magnets. In our stimulation regimen, an array of neodymium magnets with different thicknesses and field strengths were placed on an orbital shaker at 37°C/5%CO_2_ (Fig. 2A). These magnets were cycled at 42 rpm one quarter inch underneath an immobilized Aggrewell plate, which allowed us to exert a continuous magnetic field while minimizing fluid shear forces on the cells.

For our magnetic attraction experiments, we compared three types of neodymium magnets with different magnetic field strengths: ¾″×⅛″ N42 neodymium magnets, ¾″×¼″ N42 neodymium magnets, and ¾″×½″ N42 neodymium magnets (K&J Magnetics). Using a gaussmeter, we calculated that the magnetic field strengths were 0.128 Tesla, 0.2 Tesla and 0.4 Tesla respectively with the EBs placed one-quarter inch above the magnets. To model the force acting on the EBs, the cycle of magnet rotation was divided into 126 frames using a customized Matlab program (Mathworks, Natick, MA) where each frame differs by 0.05 radians. At every frame, the magnetic field at each position was modeled. The force (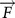) acting on each point within the wells was calculated using 
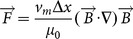
(1)where *B* is the magnetic field, *v_m_* is the total volume of the magnetic material, Δ*x* is the effective magnetic susceptibility of the RGD-Bead attached to the cell and µ_0_ is the magnetic constant in a classical vacuum as described previously. [23] The magnitude of force applied to the cells during magnetization was summarized for each of the points and averaged for each Aggrewell over 1 minute (42 cycles). From these calculations, we approximated that the force exerted on the cells was 10 piconewton (pN) at 0.128 Tesla (Fig. 2B), 20 pN at 0.2 Tesla (Fig. 2C), and 80 pN at 0.4 Tesla (Fig. 2D).

### High field strengths negatively affect short term but not long term viability

**Figure 3:**
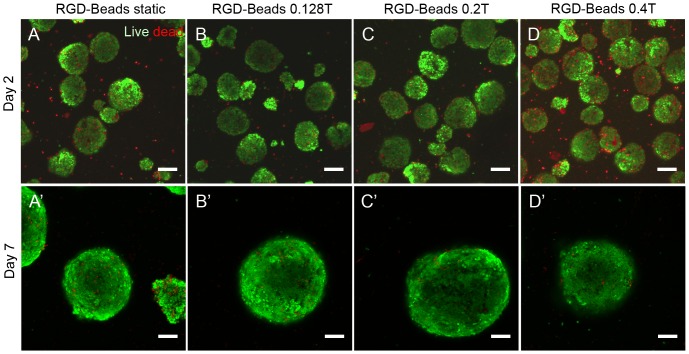
Live/dead analysis of EBs containing RGD-Beads following magnetization. Live/dead was analyzed by confocal microscopy 24 hours after magnetic attraction (A–D) or 6 days after magnetic attraction (A′–D′). Scale bar: 100 µm.

We next sought to determine whether there was any negative effect of magnetic attraction on cell viability. We performed live/dead staining on the EBs 24 hours following stimulation to examine any effects on immediate cytotoxicity, as well as on Day 7 to compare effects on long-term viability. After 24 hours, there was no difference in the number of dead cells within the EBs containing RGD-Beads maintained under static conditions (Fig. 3A), 0.128 Tesla (Fig. 3B) or 0.2 Tesla magnetic fields (Fig. 3C); however, more dead cells were stained in EBs exposed to a 0.4 Tesla magnetic field (Fig. 3D). In contrast, there was no evident effect of magnetic field strength on long-term viability in EBs with RGD-Beads (Fig. 3A′–D′). No effect on viability was observed in EBs containing AcLDL-Beads, which do not bind to integrins, or EBs without beads at any of the field strengths tested (S1 Figure).

### Mechanotransduction in EBs in response to magnetization

Integrins have been well established to play an integral role in the transmission of mechanical signals in cells [19], and their manipulation via mechanical forces has been observed to increase small molecule expression and subsequent activation of a variety of signaling cascades [13], [24]. In response to mechanical stress, cAMP levels increase, subsequently activating cAMP protein kinase (Protein Kinase A, PKA) [25], [26]. In this study, we investigated the effects of magnetically mediated strain on PKA levels in EBs. As a negative control, we also incorporated AcLDL-beads within EBs, which is a standard control for MTC because they do not specifically bind integrin [15].

**Figure 4:**
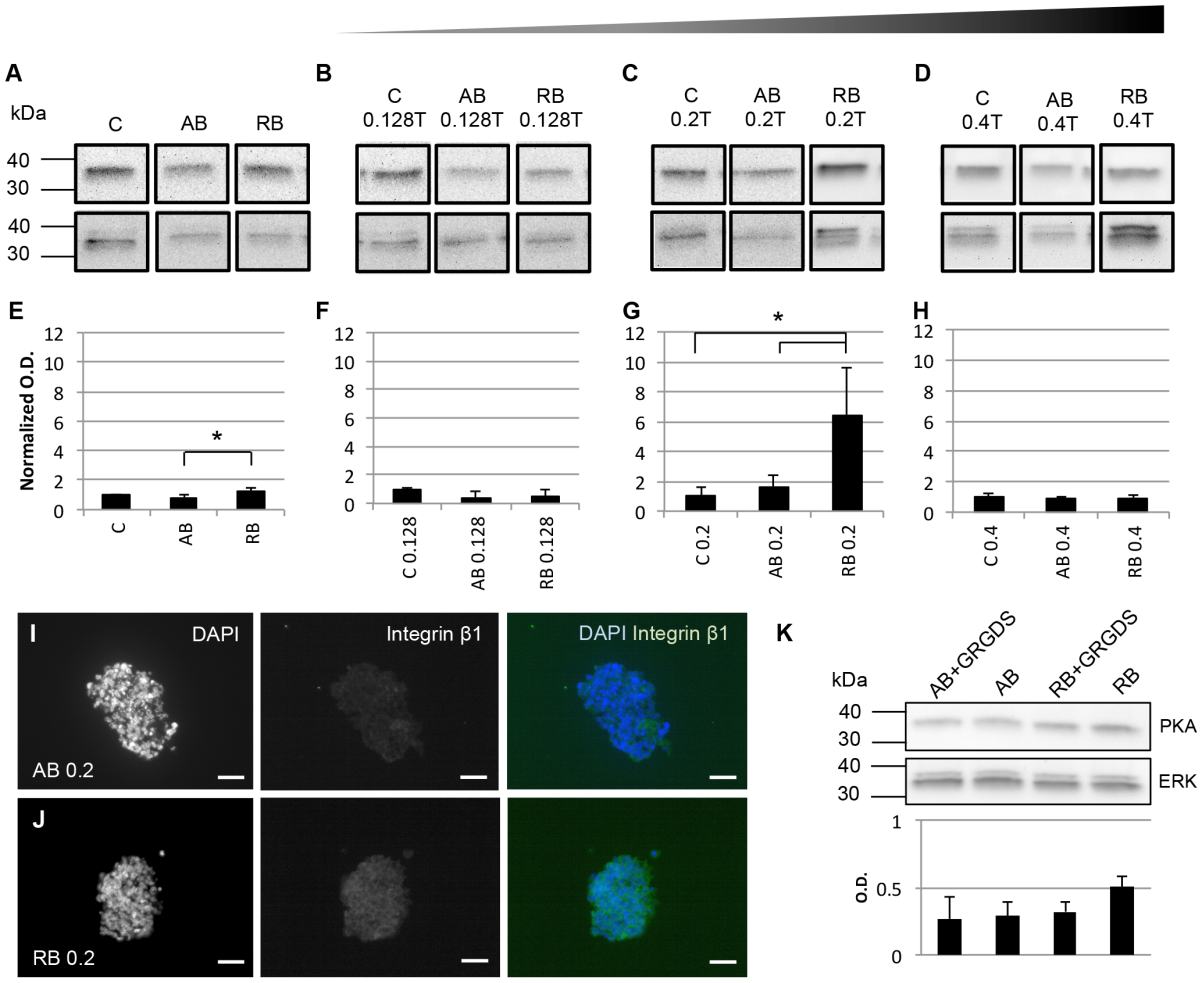
Second messenger marker expression in response to magnetic attraction. EBs incubated in static conditions (A, E) were compared to EBs exposed to 0.128 Tesla magnetic fields (B, F), 0.2 Tesla magnetic fields (C, G) and 0.4 Tesla magnetic fields (D, H). (A–D) Representative western blots under each condition. Raw western blot data is presented in S2 Figure. (E–H) Normalized PKA expression relative to ERK. (I) β1 integrin immunostaining in EBs with AcLDL-Beads and (J) RGD-Beads 24 hours following stimulation at 0.2 Tesla. Scale  = 20 µm. (K) Effect of β1 integrin inhibition by GRGDS on PKA expression. C: control, AB: AcLDL-Beads, RB: RGD-Beads. *p<0.05.

In the absence of magnetic attraction, PKA expression was observed in all samples regardless of the presence of RGD- or AcLDL-Beads (Fig. 4A, E, S2 Figure). Following one hour of exposure to a 0.128 Tesla field, there was no difference in the amount of PKA observed between samples (Fig. 4B, F, S2 Figure). As the field strength increased to 0.2 Tesla, significant differences in PKA expression could be observed between groups. While there was no difference in PKA between controls and AcLDL-Bead groups, levels increased approximately 6-fold in EBs containing RGD-Beads (Fig. 4C, G, S2 Figure). As the field strength increased to 0.4 Tesla, PKA levels decreased to the level of the unloaded and AcLDL-Bead controls (Fig. 4D, H, S2 Figure).

To further verify that the increases in PKA were the result of integrin activation by RGD-Beads, we examined the effects of magnetization on β1 integrin expression and blocking by GRGDS. Positive β1 integrin immunostaining was observed in EBs containing both AcLDL- and RGD-Beads (Fig. 4I, J). Following GRGDS inhibition, PKA levels were decreased, although not significantly, in magnetized samples containing RGD-Beads (Fig. 4K). The involvement of β1 integrin was also verified by PKA levels in AcLDL-Bead containing EBs: there was no change in PKA expression between static and magnetized groups.

**Figure 5:**
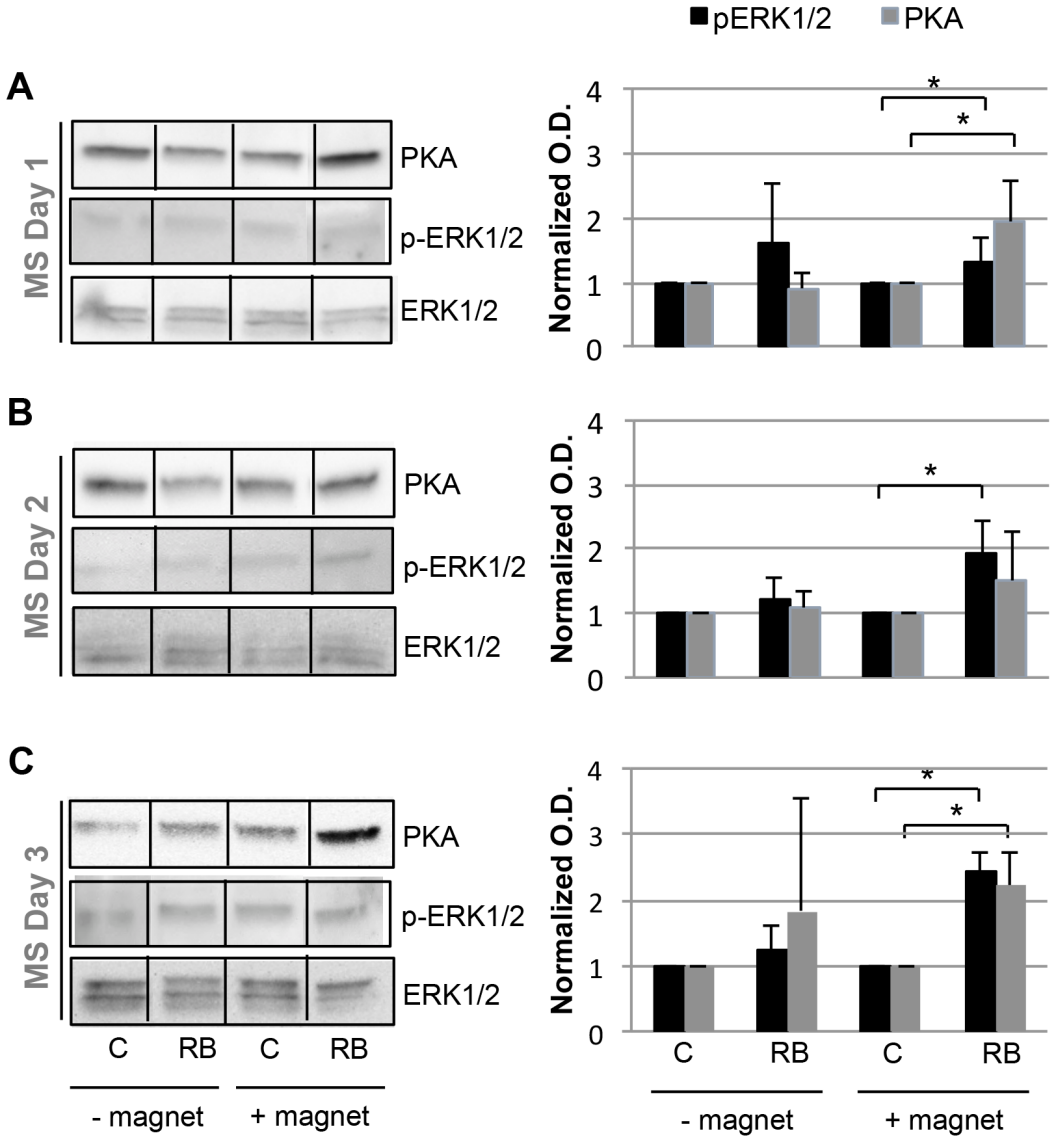
Expression of PKA and phosphorylated ERK1/2 in response to magnetic attraction at different stages of EB development. Western blot analysis of EBs stimulated on Day 1 (A), Day 2 (B) or Day 3 (C). Protein expression was normalized to ERK. MS: mechanical stimulation, C: control, RB: RGD-Beads. * *p*<0.05. Raw data is presented in S3 Figure.

Since the onset of mechanical stimulation can have different effects on ESC differentiation [5], we also investigated how the timing of magnetic mediated stress affects mechanotransduction in EBs. Regardless of whether stimulation was initiated on Day 1 (Fig. 5A, S3 Figure), Day 2 (Fig. 5B, S3 Figure) or Day 3 (Fig. 5C, S3 Figure), the expression of pERK1/2, whose expression is correlated to integrin activation [13], [27], was significantly increased following magnetic attraction. PKA expression was also significantly increased in Day 1 and 3 samples (Fig. 5A, C).

### Timing of stimulation affects cardiomyogenesis in EBs

**Figure 6:**
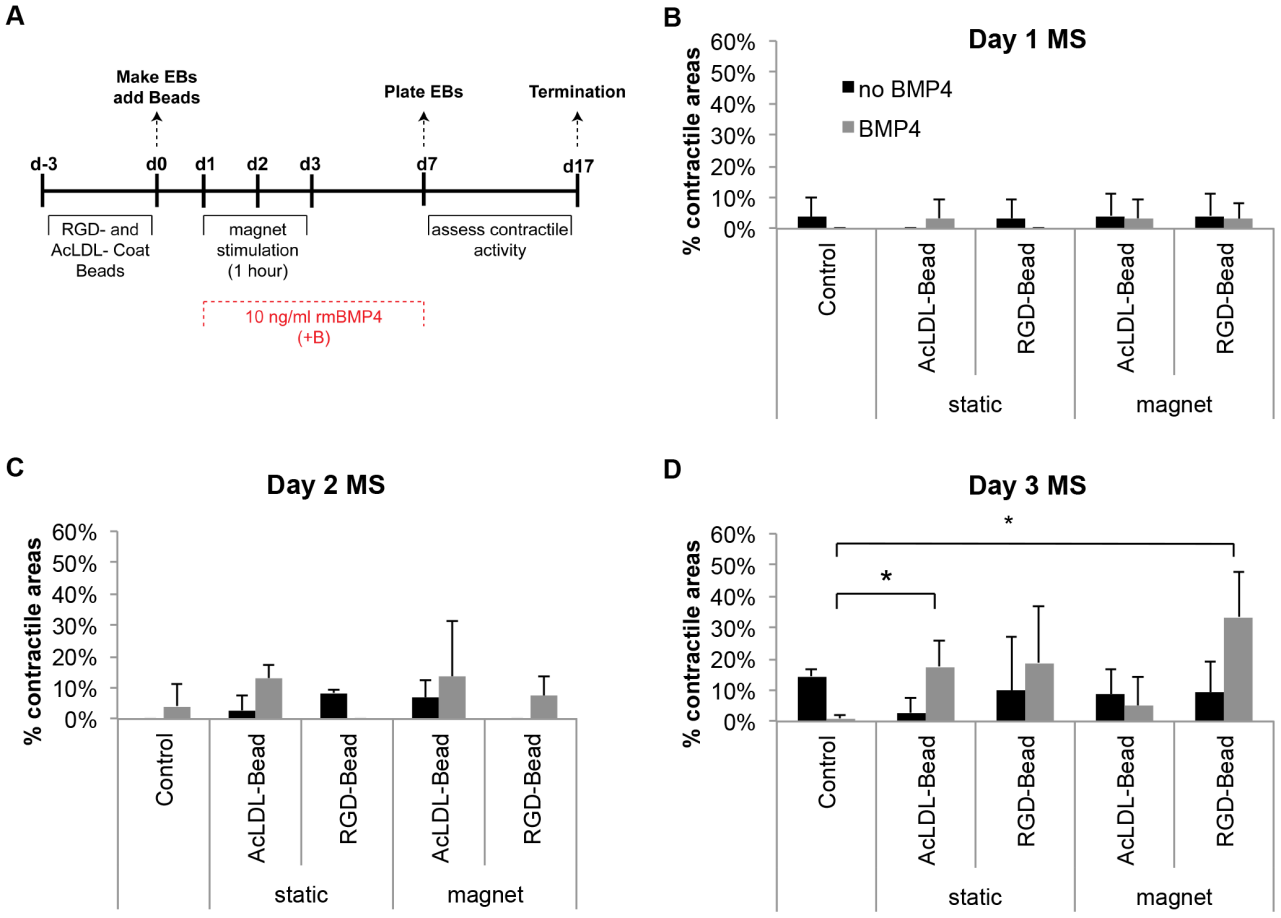
Percentage of EBs with contractile areas on Day 17. (A) Timeline of EB mechanical stimulation and culture. BMP4 (10 ng/ml) was added between Days 1–7. EBs were stimulated on Day 1 (B), Day 2 (C), or Day 3 (D) in the presence or absence of BMP4. MS: mechanical stimulation. * *p*<0.05.

Based on our observation that EBs are responsive to mechanical signals at different timepoints (Fig. 5), we sought to examine the effect of attraction timing on differentiation. ESC differentiation into cardiomyocytes has been observed following exposure to multiple types of mechanical stimuli, including shear [9] and cyclic [8], [28], [29] forces; however, the effect of magnetic attraction on cardiomyogenesis from pluripotent cells has not yet been investigated. To investigate the effect of magnetically mediated strain on ESC differentiation, we exposed the EBs to a 0.2 Tesla magnetic field, as this strain magnitude most effectively increased PKA expression while maintaining cell viability (Figs. 3, 4). EBs were stimulated for 1 hour on Day 1, Day 2 or Day 3. Since BMP4 supplementation is currently the most effective regimen for differentiating mouse ESCs into cardiomyocytes [20], we also compared the combinatorial effects of BMP4 and mechanical stimulation (Fig. 6A).

Stimulation of early EBs, specifically on Day 1 (Fig. 6B) or Day 2 (Fig. 6C), did not appear to have any significant effect on cardiomyocyte differentiation, as the percentage of contractile foci were comparable to controls with or without BMP4 supplementation. When BMP4 was added to the medium during suspension culture, Day 3-stimulated EBs (Fig. 6D) containing the RGD-Beads had significantly higher percentages of contractile foci compared to controls by Day 17 (33%±14% versus 4%±7%). The presence of beads alone also appeared to have an effect on contractile activity compared to controls; however, only AcLDL-Bead-containing EBs had significantly higher levels than controls (18%±8%). These trends were not observed when BMP4 was omitted from the medium (Fig. 6D).

**Figure 7:**
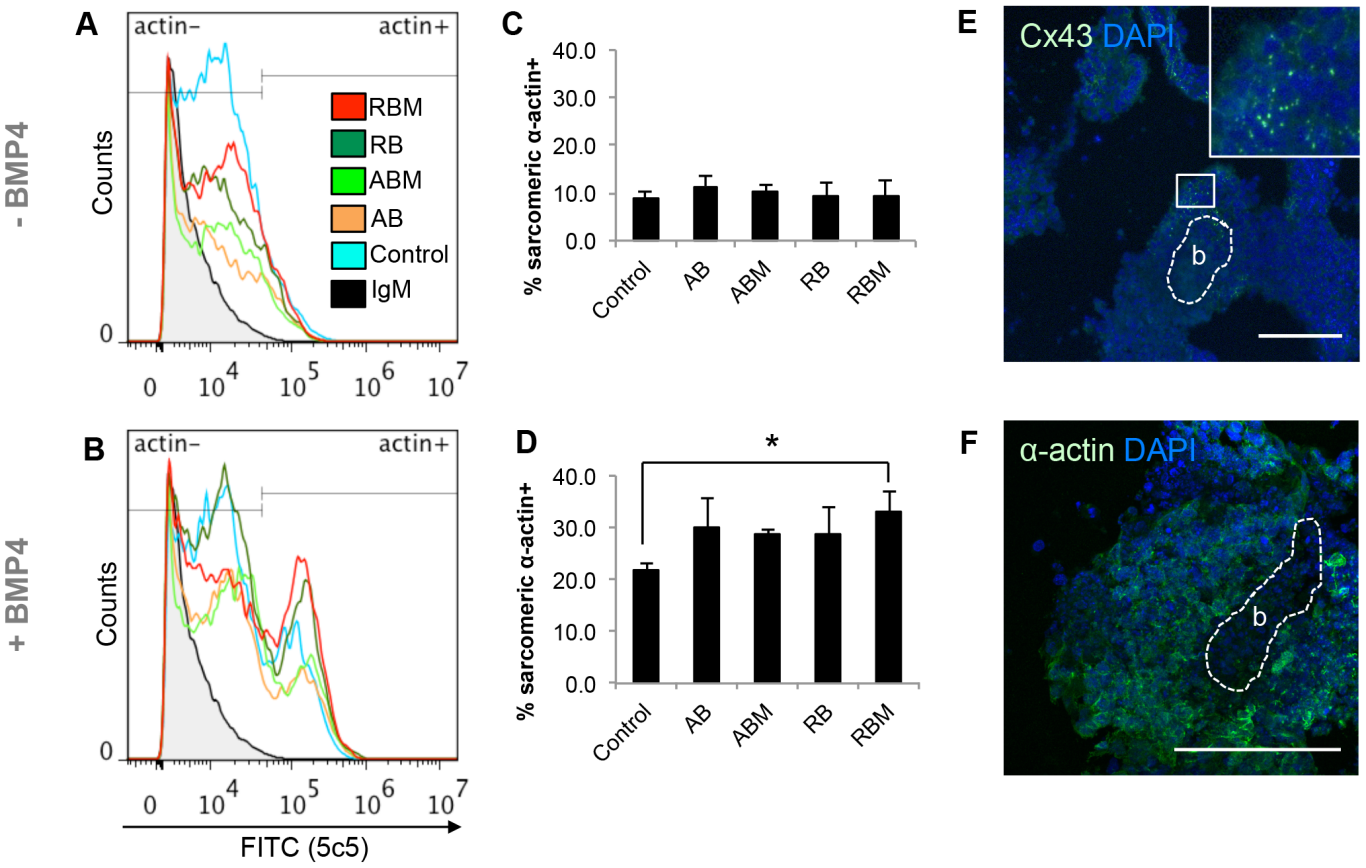
Sarcomeric α-actin expression in Day 3-stimulated EBs. Histogram representation of sarcomeric α-actin+ cells in Day 18 EBs without BMP4 (A) and with BMP4 (B). (C–D) Percent of sarcomeric α-actin cells in EBs without (C) and with (D) BMP4 supplementation. (E) Cx43 (inset: magnification of cx43 between cells) and (F) sarcomeric α-actin immunostaining of Day 3-stimulated EBs on Day 18. “b”: location of beads within confocal micrograph. AB: AcLDL-Beads, RB: RGD-Beads, M: magnetized. *p<0.05. Scale bar: 20 µm.

Considering the difference in contractile activity, we further examined the expression of sarcomeric α-actin within the Day 3-stimulated EBs on Day 18 by FACS analysis and immunostaining. For the former, in the presence of BMP4 there was a shift in fluorescence intensity in all EBs (Fig. 7B, S4 FigureA) compared to those which were not exposed to BMP4 (Fig. 7A, S4 FigureB). In the absence of BMP4, there were no significant differences between the control and EBs containing either AcLDL-Beads or RGD-Beads (Fig. 7C). With BMP4 added to the medium, there were significantly more sarcomeric α-actin positive cells in stimulated EBs containing RGD-Beads (33.2%±3.8%) compared to unloaded controls (22.0%±1.1%)(Fig. 7D). While the presence of the beads alone appeared to increase the percentages of sarcomeric α-actin-expressing cells, there was no statistical increase compared to controls (Fig. 7D). Connexin 43 (Fig. 7E) and sarcomeric α-actin (Fig. 7F) immunostaining was observed sections of Day 18 EBs released from gelatin-coated plastic. Expression was observed in the vicinity of beads (labeled as “b”), which could not be observed directly by confocal microscopy but appeared as a shadow in the micrographs.

## Discussion

In this study, we developed a unique method to expose a larger population of ESCs within an EB to mechanical strain. We demonstrated that RGD-Beads, such as those used for MTC, can be incorporated into the interior of the EB and bind to cells within that EB. By adjusting the thickness of the magnets, and consequently the magnetic field strength, we can take advantage of the RGD-integrin interactions to induce strain on the individual cells without compromising viability. A complication that occurs with current mechanical stimulation regimens is that ESCs experience non-uniform forces, which produce the same heterogeneous cell population that would occur naturally without stimulation. Immobilization of RGD-Beads within the EB interior has the potential to reduce this variability. In addition, this process does not impact differentiation protocols that would depend on suspension culture during downstream processing.

A benefit of this mechanical stimulation regimen is that it is a cost-effective method to present a permanent magnetic field to cells in culture. The magnetic field strength can be easily adjusted by altering the strength of the neodymium magnets, and the force acting on the cells in response to magnetization can be deduced using the equation developed by Zborowski *et al* (1999) for a magnetized cell in a quadrupole field (Equation 1) [23]. Overall, our results confirm previous findings that ESCs are responsive to integrin-mediated forces, and these forces lead to outside-in signaling cascades that can direct cell fate [16], [17]. These forces can be assessed by the expression of small molecules and proteins, such as PKA and pERK1/2, which are well established to be directly regulated by mechanical stimulation (Figs. 5,6) [13], [24], [25]. Consequently, the same methods that are used to study mechanical signal transmission in differentiated cells can be applied to pluripotent stem cells.

In general, there is limited information about the amount of force required by cells to activate signaling or respond to external stress. This has been most recently addressed by Wang *et al* (2013), whom have developed a Tension Gauge Tether (TGT) technique to quantify the amount of force required to activate integrin and Notch signaling cascades in immortalized CHO cells [30]. The authors reported that Notch signaling was activated by less than 12 pN force, and that cells apply approximately 40 pN force to single integrin-ligand adhesions. Forces above 40 pN result in stress fiber formation *in vitro*. Based on our model, 0.2 Tesla magnetic fields exert approximately 20 pN force on the ESCs. Doubling the magnetic field to 0.4 Tesla exerts almost 80 pN force, which negatively affects signal transduction and viability. While we would predict that 20 pN would be too low to transmit mechanical signals, our results suggest that this magnitude is sufficient to activate integrins. There are a few possible explanations for this discrepancy: (1) there may be differences in force sensitivity between differentiated and pluripotent stem cells or (2) there may be other forces experienced by the cells within the EB, such as shear forces between cells, that are not adequately represented by either model. In the future, a better understanding of the range of forces and force strengths being experienced during ESC aggregation may clarify these questions.

Cell-matrix interactions, and their manipulation, have been widely observed to play important roles in directing pluripotent cell fate [17], [31]–[35]. Considering mechanical stimulation can direct cardiomyogenesis from pluripotent cells [5], we asked whether magnetic attraction can similarly direct differentiation. Timing the onset of mechanical stimulation can have drastic effects on fate determination in regards to cardiovascular phenotypes. While short term applications of cyclic strain between days 4 and 7 during EB development increase the percentage of differentiated cardiomyocytes [6]–[8], early and prolonged exposure maintains pluripotency [36], [37]. Our results similarly suggest that cardiomyogenesis in EBs is most effective when mechanical stimulation is initiated later during EB development, around Day 3. This was made evident by maintained contractile activity at Day 17 and significantly increased sarcomeric α-actin expression (Figs. 6,7). Interestingly, this is only observed when BMP4, which is the most effective morphogen for mESC differentiation into cardiomyocytes, is supplemented to culture medium. Considering that Brachyury expression, a marker for mesendoderm [38], peaks around Day 3 and 4 of EB development [39]–[41], this would suggest that pre-differentiation of EBs towards mesodermal fates prior to the onset of mechanical stimulation is optimal for cardiomyogenesis *in vitro.* Therefore, while mechanical stimulation by magnetization is alone not sufficient to drive differentiation, it can be combined with other protocols to enhance cardiomyocyte differentiation.

While BMP4 supplementation to mouse EBs is well established to promote cardiomyogenesis, we only observed modest increases in contractile activity. In contrast, our sarcomeric α-actin expression data (Fig. 7) suggest that BMP4 is doubling the percentage of cardiomyocytes compared to EBs cultured in the absence of BMP4. A possible explanation for this is the Aggrewell plate culture system. Previous studies have demonstrated that the EB aggregation and culture method can have profound effects on downstream contractile activity, even when the medium components are constant [22]. To the best of our knowledge, this is the first study to examine cardiomyogenic differentiation in EBs from the R1 mouse ES cell line using Aggrewell plates in the presence of BMP4. Loading beads into EBs using a different aggregation method, such as hanging drop, may further enhance contractile activity and exacerbate the differences between groups.

Another interesting finding from these experiments is the effect of the EB microenvironment on guiding cell fate. Previous studies have observed that the presence of microparticles alone within the EB interior can increase the expression of mesodermal markers [42]. We also found that the presence of the paramagnetic beads alone had some effect on both contractile activity and sarcomeric α-actin expression (Figs. 6D, 7B). This suggests that microparticle presence activates either a biological or physical response in cells within the EB that can affect differentiation; however, based on our results, only in the presence of magnetically mediated strain will lineage commitment be significantly enhanced compared to controls. In the future, further studies into the mechanism behind these EB interactions may be necessary.

To date, there are few studies that have been able to investigate the effects of early mechanical stimulation on the terminal phenotype of ESCs. Often this issue is due to logistics: there is a minimum amount of time required to allow EBs to attach to culture surfaces and also acclimate to new environments prior to exposure to stress. While this study primarily focused on cardiomyocyte differentiation, earlier exposure to magnetization (Days 1–2) may be driving differentiation into other phenotypes. This is suggested by preliminary data, in which EBs magnetized on Day 1 express significantly less SSEA-1, a marker for mouse ESC pluripotency, by Day 7 compared to EBs stimulated on Day 2 or 3 (data not shown). Further investigation into the effects of integrin-RGD interactions, and how they change during the first few days of EB differentiation, will be useful for tailoring magnetic attraction to derive specific differentiated cells.

## Conclusions

Our data showed that magnetically mediated strain applied by RGD-immobilized paramagnetic beads activates mechanochemical signaling in murine EBs. We also observed that ESC response to strain is dependent on the magnetic field strength, as high field strengths can apply too much force on the cells, resulting in cell death. Magnetically mediated strain can also be used to direct differentiation of mESCs into cardiomyocytes; however guiding the mesodermal commitment of mESCs prior to the onset of stimulation is required. Conceivably, this system may be tailored to derive other cell types that are similarly influenced by mechanical forces by altering the medium supplements and timing accordingly.

## Supporting Information

S1 Figure **Live/dead imaging of EBs with or without AcLDL-Beads.** Live cells (green) and dead cells (red) were observed in controls and EBs with AcLDL-beads 24 hours after magnetization and 6 days after magnetization on Day 1. Scale  = 100 µm.
(TIF)

S2 Figure **Representative western blots of PKA and ERK expression following magnetic attraction.** N = 2 for each group. Blots were repeated for an additional two samples for a total N = 4. Highlighted blots are presented in Fig. 4.
(TIF)

S3 Figure **Representative western blots of PKA and pERk1/2 expression following magnetic attraction on Days 1-3.** Shaded data is not discussed in this manuscript.
(TIF)

S4 Figure **FACS dot plot data of sarcomeric α-actin-expressing population on Day 18.** (A) EBs without supplemented BMP4. (B) EBs with supplemented BMP4
(TIF)
