# Supplementary figures and images for: Paramagnetic Beads and Magnetically Mediated Strain Enhance Cardiomyogenesis in Mouse Embryoid Bodies
Source: PLoS One. 2014 Dec 12;9(12):e113982. doi: 10.1371/journal.pone.0113982 (PMC4264692; doi:10.1371/journal.pone.0113982)

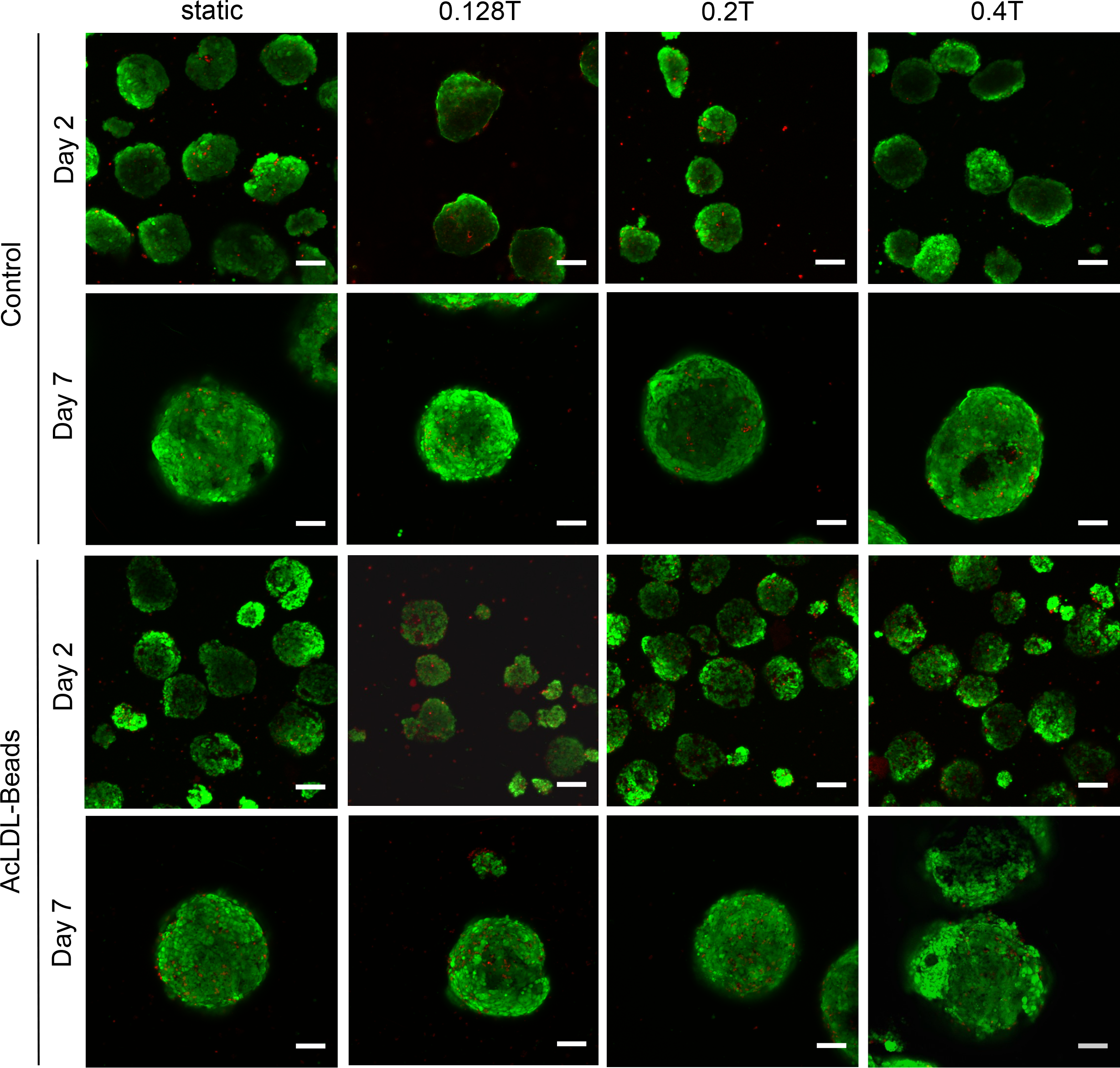

Supplement: S1 Figure — Live/dead imaging of EBs with or without AcLDL-Beads. Live cells (green) and dead cells (red) were observed in controls and EBs with AcLDL-beads 24 hours after magnetization and 6 days after magnetization on Day 1. Scale = 100 µm. (TIF) [file pone.0113982.s001.tif]

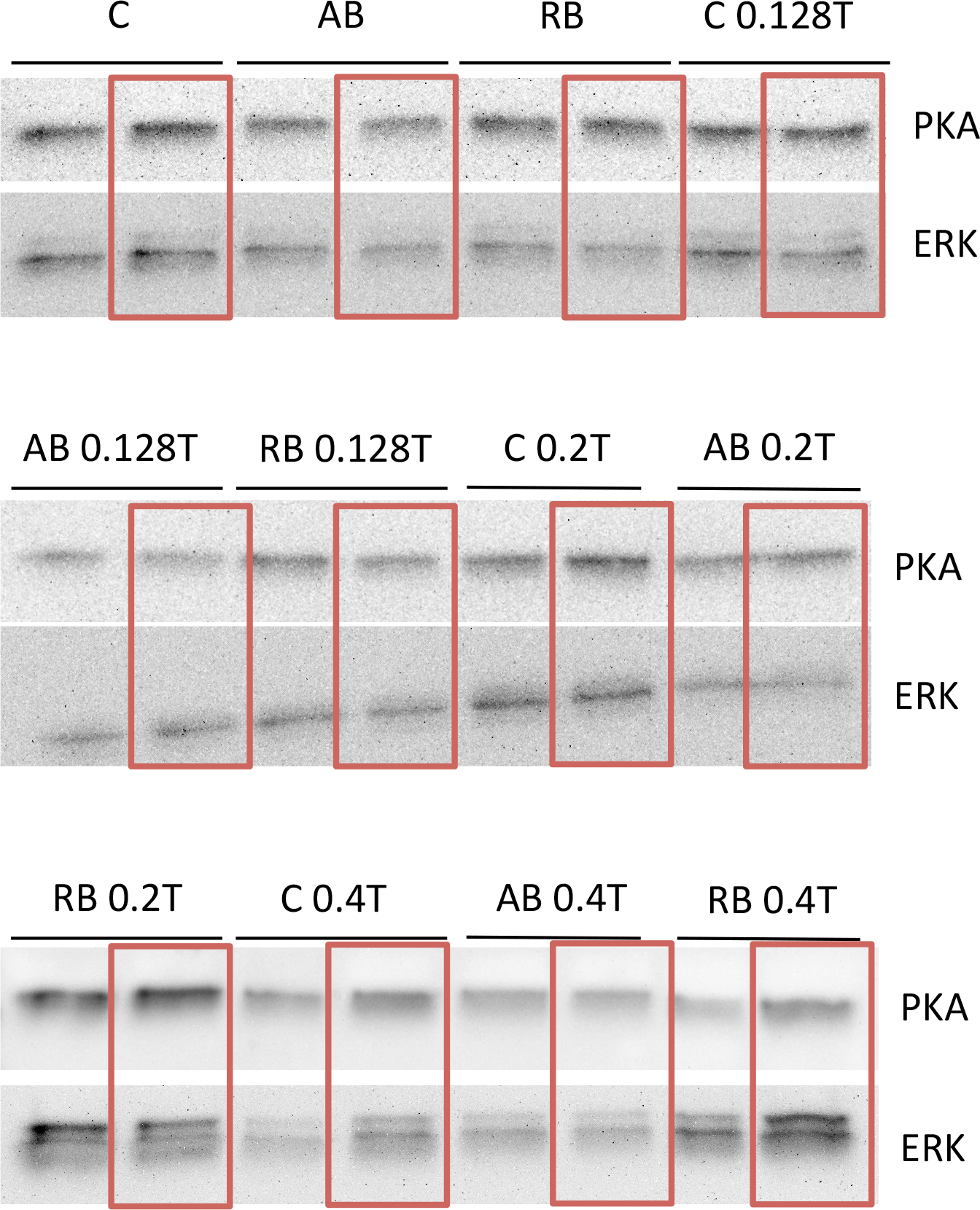

Supplement: S2 Figure — Representative western blots of PKA and ERK expression following magnetic attraction. N = 2 for each group. Blots were repeated for an additional two samples for a total N = 4. Highlighted blots are presented in Fig. 4. (TIF) [file pone.0113982.s002.tif]

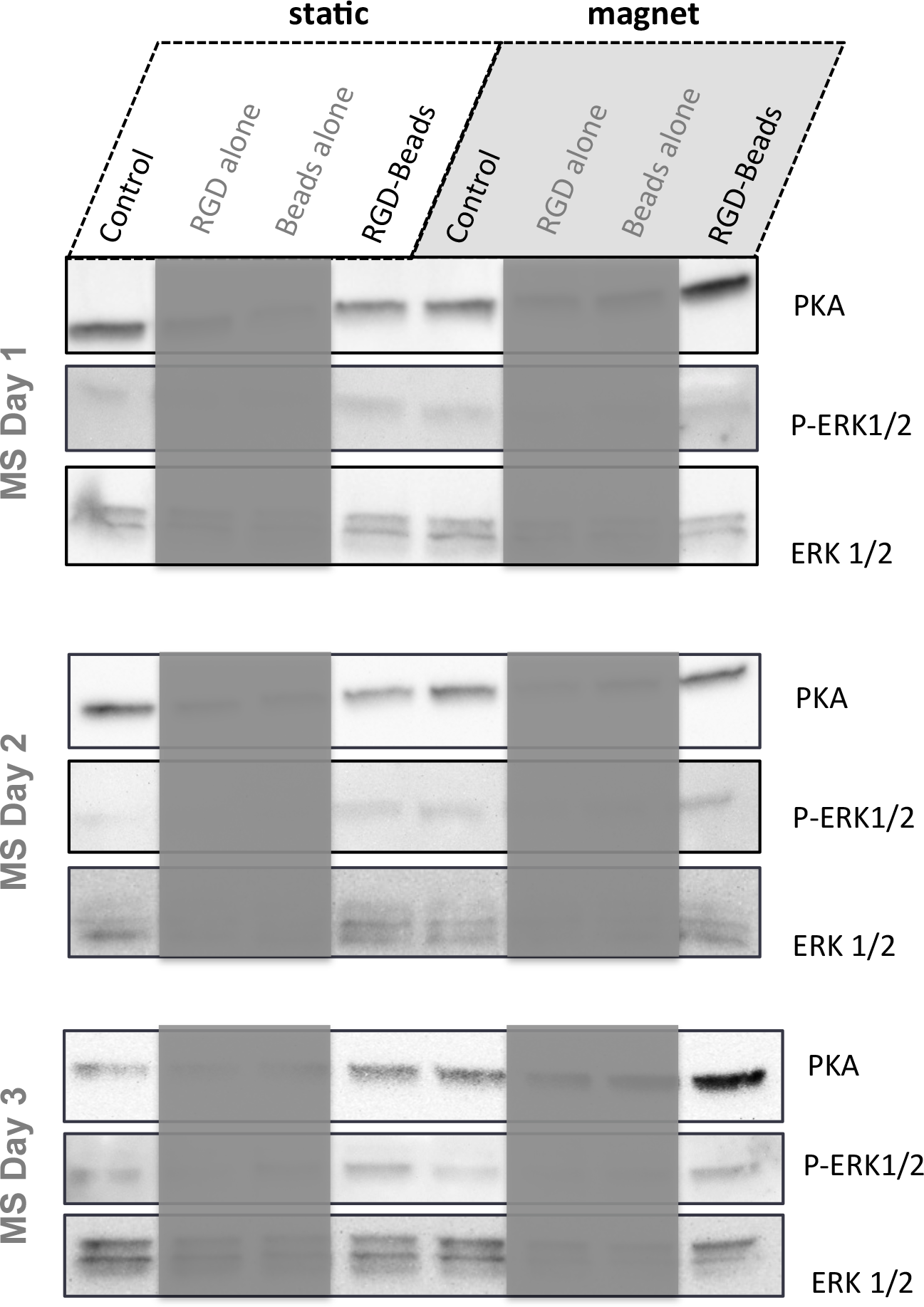

Supplement: S3 Figure — Representative western blots of PKA and pERk1/2 expression following magnetic attraction on Days 1-3. Shaded data is not discussed in this manuscript. (TIF) [file pone.0113982.s003.tif]

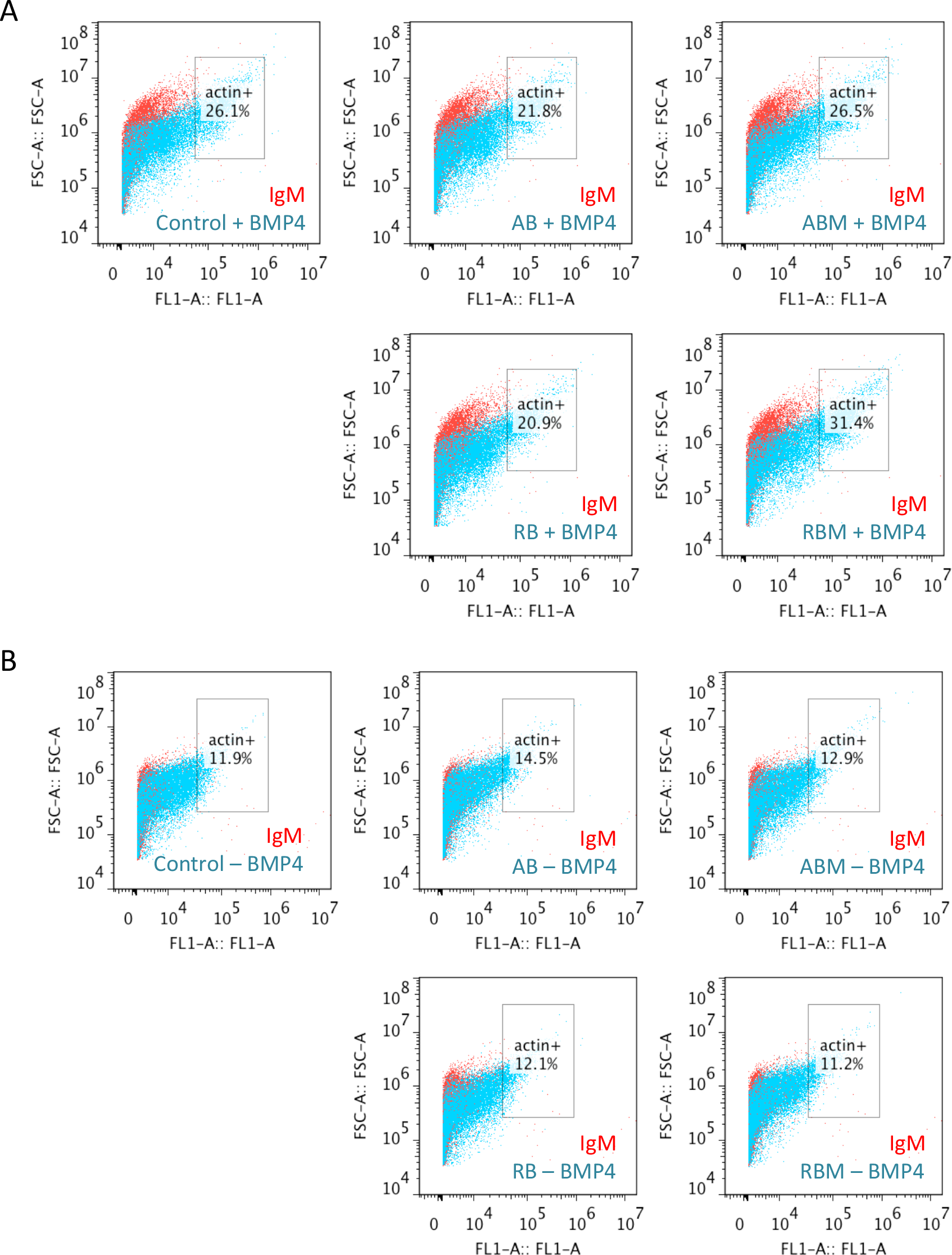

Supplement: S4 Figure — FACS dot plot data of sarcomeric α-actin-expressing population on Day 18. (A) EBs without supplemented BMP4. (B) EBs with supplemented BMP4 (TIF) [file pone.0113982.s004.tif]
